# 151. Detection of highly pathogenic avian influenza A(H5N1) in contrived nasal swab specimens using commercial molecular influenza A tests

**DOI:** 10.1093/ofid/ofaf695.053

**Published:** 2026-01-11

**Authors:** Anuradha Rao, Leda Bassitt, Heather Bowers, Courtney Sabino, Evelyn K Williams, Julie Sullivan, Emily B Kennedy, Jacob Khouri, Pamela A Miller, Eric Lai, Raymond F Schinazi, Beverly Rogers, Wilbur A Lam, Nira Pollock, Gregory L Damhorst

**Affiliations:** Emory University School of Medicine, Atlanta, Georgia; Emory University School of Medicine, Atlanta, Georgia; Emory University, Atlanta, Georgia; Emory University, Atlanta, Georgia; Emory University School of Medicine, Atlanta, Georgia; Emory University, Atlanta, Georgia; OOMVELT, Lakewood, Ohio; Breton Highlands Consulting, Sand Springs, Oklahoma; Echo Consulting, Excelsior, Minnesota; VentureWell, San Diego, California; Emory University School of Medicine, Atlanta, Georgia; Emory University School of Medicine, Atlanta, Georgia; Emory University School of Medicine/Georgia Institute of Technology, Atlanta, GA; Boston Children's Hospital, Boston, MA; Emory University, Atlanta, Georgia

## Abstract

**Background:**

At least 70 human cases of highly pathogenic avian influenza (HPAI) A H5N1 (i.e., "bird flu") have been reported in the U.S. since 2024, primarily among individuals with exposure to dairy cattle or poultry. These cases, along with the rapid transmission of bird flu among U.S. wild bird, poultry, and cattle populations, warrant testing of available influenza A diagnostics for their ability to detect 2024 H5N1 strains. We previously tested 12 lateral flow assays (LFAs) and 5 point-of-care (POC) nucleic acid amplification tests (NAATs) with influenza A(H5N1) clade 2.3.4.4b (https://doi.org/10.1101/2025.04.15.25325613) as part of pandemic preparedness assessment. Here we expand the analysis by testing six additional FDA-cleared small footprint, sample-to-answer NAATs. The tests are anonymized pending authorization from each company.

**Table 1:**
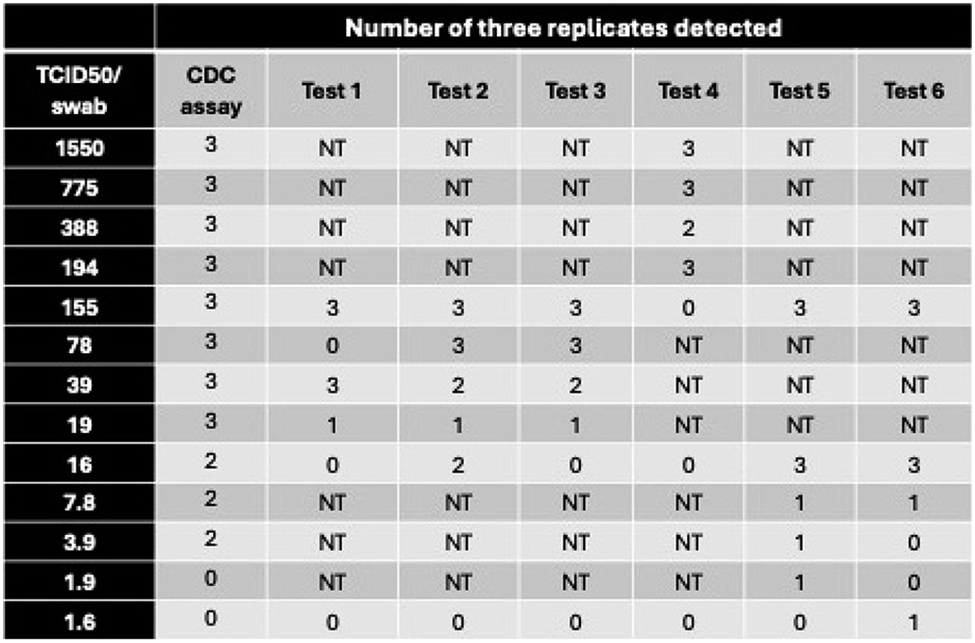
Relative sensitivity of six FDA-cleared NAAT assays for detection of a U.S. bovine 2024 HPAI H5N1 strain. All data were generated using the inclusivity protocol with heat-inactivated 2024 HPAI H5N1. The CDC Human Influenza Virus Real-time RT-PCR Diagnostic Panel, Influenza A/B Typing Kit was performed as a Research Use Only application. Samples were tested in triplicate, and the lowest concentration detected as 3/3 positive is highlighted in bold. Tests 1-6 are anonymized pending permissions. NT: Not tested.

**Methods:**

We performed testing with specimens contrived with heat-inactivated 2024 H5N1 clade 2.3.4.4b genotype B3.13 (bovine, *BEI,* NR-59872) by spiking negative nasal swab matrix with a known quantity of virus and producing a dilution panel. Fifty microliters of contrived specimen was added directly to a swab and the swab added to 3.0 mL UTM; each dilution was tested in triplicate for each of six assays according to respective instructions for use. The lowest dilution producing 3/3 positive results was recorded as the detection limit. The dilution panel was also tested with the CDC H5 genotyping assay as a reference. Prior work assessing LFAs (live virus) and NAATs used the same methods.

**Results:**

The six FDA-cleared molecular tests consistently detected 2024 H5N1 (B3.13) at 16 to 775 TCID50/swab (Table 1). In comparison, prior testing of POC molecular assays demonstrated detection at 1.55 to 7.75 TCID50/swab. Prior testing with live virus showed that 11 of 12 LFAs detected 2024 H5N1 with sensitivity ranging from 78 to 1550 TCID50/swab with one outlier that was above 1550 TCID50/swab.

**Conclusion:**

Six FDA-cleared small footprint, sample-to-answer molecular influenza A tests consistently detected a U.S. 2024 H5N1 strain in contrived nasal swab specimens. In the event of human-to-human transmission, clinical performance and optimal sample types would need to be established for these and other clinical influenza A diagnostics.

**Disclosures:**

All Authors: No reported disclosures

